# The Multifaceted Role of TGF-β in Gastrointestinal Tumors

**DOI:** 10.3390/cancers13163960

**Published:** 2021-08-05

**Authors:** Fabio Sabbadini, Monica Bertolini, Serena De Matteis, Domenico Mangiameli, Serena Contarelli, Silvia Pietrobono, Davide Melisi

**Affiliations:** 1 Digestive Molecular Clinical Oncology Research Unit, Department of Medicine, University of Verona, 37134 Verona, Italy; fabio.sabbadini@univr.it (F.S.); monica.bertolini@univr.it (M.B.); serena.dematteis2@unibo.it (S.D.M.); domenico.mangiameli@univr.it (D.M.); serena.contarelli@univr.it (S.C.); silvia.pietrobono@ittumori.it (S.P.); 2 Department of Experimental, Diagnostic and Specialty Medicine, AlmaMater Studiorum, University of Bologna, 40126 Bologna, Italy; 3 Experimental Cancer Medicine Unit, Azienda Ospedaliera Universitaria Integrata di Verona, 37134 Verona, Italy

**Keywords:** TGF-β, gastrointestinal tumors, EMT, targeted therapy, immune plasticity

## Abstract

**Simple Summary:**

The transforming growth factor β signaling pathway elicits a broad range of physiological re-sponses, and its misregulation has been related to cancer. The secreted cytokine TGFβ exerts a tumor-suppressive effect that counteracts malignant transformation. However, once tumor has developed, TGFβ can support tumor progression regulating epithelial to mesenchymal transition, invasion and metastasis, stimulating fibrosis, angiogenesis and immune suppression. Here we review the dichotomous role of TGF-β in the progression of gastrointestinal tumors, as well as its intricate crosstalk with other signaling pathways. We also discuss about the therapeutic strate-gies that are currently explored in clinical trials to counteract TGF-β functions.

**Abstract:**

Transforming growth factor-beta (TGF-β) is a secreted cytokine that signals via serine/threonine kinase receptors and SMAD effectors. Although TGF-β acts as a tumor suppressor during the early stages of tumorigenesis, it supports tumor progression in advanced stages. Indeed, TGF-β can modulate the tumor microenvironment by modifying the extracellular matrix and by sustaining a paracrine interaction between neighboring cells. Due to its critical role in cancer development and progression, a wide range of molecules targeting the TGF-β signaling pathway are currently under active clinical development in different diseases. Here, we focused on the role of TGF-β in modulating different pathological processes with a particular emphasis on gastrointestinal tumors.

## 1. Introduction

Transforming growth factor β (TGF-β) is a secreted cytokine that regulates proliferation, migration, and differentiation of a multitude of cell types. TGF-β has a key role in embryonic development and homeostasis, inflammation, and tissue repair [1]. It elicits a broad range of context-dependent physiological responses, and alterations in this signaling have been related to many diseases, including cancer. During the early stages of tumorigenesis, TGF-β acts as a tumor suppressor by inducing cytostatic effect and apoptosis in normal and pre-malignant cells. However, once the tumor has developed, TGF-β functions as a tumor promoter triggering epithelial to mesenchymal transition, which leads to increased invasiveness and metastasis promotion [2]. TGF-β also supports tumor progression by stimulating fibrosis, angiogenesis, and immune suppression [3,4,5]. This dichotomy in TGF-β functions remains a fundamental roadblock to effectively targeting the TGF-β pathway for treating human cancers.

The gastrointestinal (GI) tract is the main responsible a couple of organs for cancer-related deaths compared to any other organ system through the body. Alterations in TGF-β signaling have been well documented in different types of GI tumors [6,7,8], and increased secretion of TGF-β has been associated with a poor prognosis in patients at advanced stages [9]. In this review, we summarize the current knowledge of the TGF-β signaling pathway in GI tumors, focusing on the main regulatory mechanisms beyond its activation and on the signaling interplay between TGF-β and tumor suppressors or promoters involved in the variety of cellular responses exerted by TGF-β during GI progression. Therapeutic approaches to counteract TGF-β functions will be discussed as well.

## 2. TGF-β Secretion and Activation

TGF-β is part of a protein family of cytokines and growth factors composed of 32 genes, which can be divided into two subgroups: the first group is represented by TGF-βs, Nodal, and Activin, and the second by bone morphogenetic proteins (BMPs) [10]. In mammals, there are three different isoforms of TGF-β (TGF-β1, TGF-β2, and TGF-β3), which are characterized by 75% homology and similar signaling activities but show variable expression in different cell types and/or tissues upon various cellular stresses and depending on physio-pathological conditions [11]. While TGF-β1 is mainly induced by signals promoting growth and proliferation, TGF-β2 and TGF-β3 expression increases after differentiation stimuli and growth arrest signals. In general, TGF-β1 is the most abundant and ubiquitously expressed isoform [12].

TGF-β is secreted in an inactive form as a homodimeric pro-peptide, with an N-terminal sequence represented by the mature cytokine and a C-terminal sequence named latency-associated peptide (LAP) that prevents TGF-β activation [13]. In the trans-Golgi, the pro-domain LAP is glycosylated and cleaved from the mature cytokine by furin protease but remains non-covalently attached, forming the small latent complex (SLC). Dimers of TGF-β:LAP associate with the latent TGF-β binding protein (LTBP) to form the large latent complex (LLP), which is sequestered and stored within the extracellular matrix (ECM) [14]. While LAP prevents TGF-β activation, LTBP serves as a chaperone for TGF-β folding and secretion into the extracellular space and can mediate the association of TGF-β with ECM proteins. Activation of TGF-β is achieved by proteolytic cleavage of LAP or by structural deformation of LLP due to a mechanical force generated by the binding of integrins to an arginine-glycine-aspartate (RGD) motif in LAP, resulting in the release of mature and active TGF-β dimer [15,16,17].

Active TGF-β has a shorter half-life than latent TGF-β and, in the absence of any association with its receptor, it is rapidly cleared from the extracellular space [18]. Thus, activation of latent TGF-β tightly allows the spatial and temporal regulation of TGF-β signaling.

## 3. The TGF-β Signaling Pathway

TGF-β signals through single-pass transmembrane receptors with serine/threonine kinase activity to transduce signal inside the cell. There are three types of TGF-β receptors: 7 type I activin-like receptors (type I) (ALK 1–7) (TβRI), 5 type II receptors (TβRII), and 2 type III receptors (betaglycan and endoglin) (TβRIII) [19]. TβRI, known as ALK5, and TβRII function as receptors for TGF-β ligands. They possess similar structural and functional properties, with a small disulphide-rich ectodomain, a single-spanning transmembrane domain, and a cytoplasmatic serine-threonine kinase domain. Due to its dimeric structure, TGF-β can interact concurrently with both TβRI and TβRII. Binding of TGF-β ligand to TβRII leads to the recruitment of TβRI to form a heteromeric complex with subsequent TβRII-mediated transphosphorylation and activation of TβRI, which propagates signals to the intracellular effectors [20,21,22,23,24,25]. Type III receptors, which are characterized by a longer extracellular domain lacking the kinase signaling motif, also contribute to the activation process functioning as accessory co-receptors [26]. 

The main mediators of TGF-β family signaling are proteins belonging to the SMAD family, an acronym obtained by merging *Caenorhabditis elegans Sma* genes and the *Drosophila Mad*, *Mothers against decapentaplegic*. SMAD proteins transduce signals into the nucleus leading to the activation of different target genes involved in proliferation, differentiation, chemotaxis, and immune modulation [27]. Depending on their functional role, SMAD proteins are divided into three different groups: the receptor-activated SMADs (R-SMAD) (e.g., SMAD1, SMAD2, SMAD3, SMAD5, and SMAD8) that are phosphorylated by type I receptor and serve to transmit signals into the nucleus; the inhibitory SMADs (e.g., SMAD6 and SMAD7) that suppress receptor and SMAD signaling, acting in a negative feedback loop; and the common-mediator SMADs (e.g., SMAD4) that act as co-factors for R-SMAD [28]. Upon TGF-β ligand binding, SMAD2 and SMAD3 undergo phosphorylation on C-terminal serine residues, dissociate from TβRI, and form a heterotrimeric complex with the common mediator SMAD4. This trimeric complex migrates into the nucleus, where it activates or represses the transcription of hundreds of genes involved either in tumor suppression or tumor progression [29,30]. The interaction of SMADs with co-activators and co-repressors is required for efficient DNA binding and is dependent on both intracellular and extracellular stimuli, contributing to the tissue- and context-dependent effects of TGF-β [2].

Besides the canonical SMAD-dependent signaling, activation of TGF-β receptors also results in the induction of several SMAD-independent pathways, including the mitogen-activated protein kinases (MAPK), c-Jun N-terminal Kinase (JNK)/p38 MAPK, Extracellular Signal-Regulated Kinases (ERKs), phosphatidylinositol-3 kinase (PI3K)/Akt, Rhodopsin (Rho) family GTPases involved in actin cytoskeletal changes and tight-junction resolution, and TGF-β-activated kinase-1 (TAK1) that modulates cell survival and inflammatory responses [31,32,33]. These SMAD-independent pathways can directly impact the activity of R-SMADs. For instance, AKT regulates SMAD3 activity by sequestering it in the cytoplasm, independent of its kinase activity [34], while ERK can activate SMAD2/3 through phosphorylation [35]. The SMAD-independent transcriptional and translational responses may crosstalk with SMAD-mediated activities to suppress or promote tumor progression [36].

**Figure 1:**
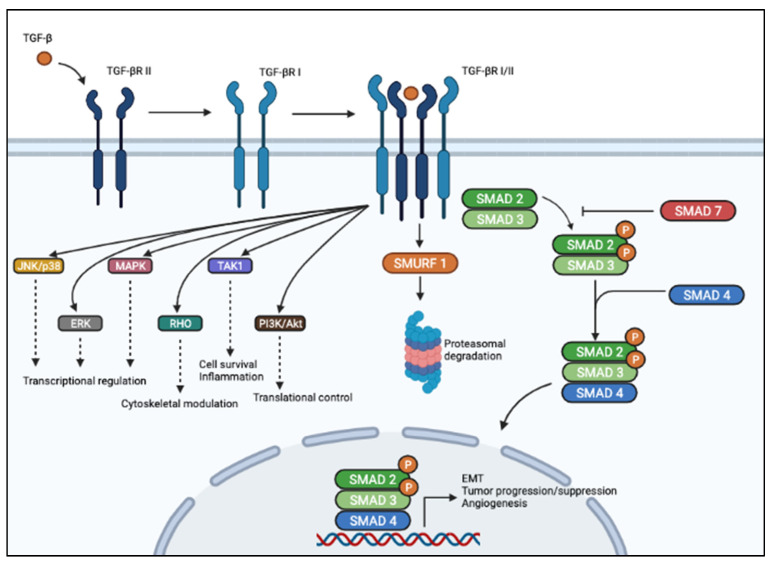
Schematic representation of TGF-β signaling pathway. In the canonical pathway (right), TGF-β ligand binds to membranous TBβRII homodimers with high affinity. This figure was created with BioRender.com, accessed on 4 June 2021.

TGF-β signaling is regulated at multiple levels. Mainly, the inhibitory SMAD7 inhibits TGF-β signaling by interacting with TβRI and with R-SMADs [37], while SMAD ubiquitination regulatory factor (Smurf)-1 dynamically regulates TGF-β signaling by inducing TβRI degradation [38]. A schematic diagram of the TGF-β signaling pathway is provided in Figure 1.

This allows heterodimerization with TβRI homodimers, activation of the TβRI kinase domain, and signal transduction via phosphorylation of the C-terminus of SMAD2 and SMAD3. SMAD2/3 dimer forms a heterotrimeric complex with SMAD4, which translocates into the nucleus and activates or represses transcription of different genes regulating cell growth, apoptosis, motility, epithelial-mesenchymal transition, angiogenesis, etc. In the non-canonical pathway (left), TGF-β signaling activates SMAD-independent pathways such as PI3K/AKT, MAPK, ERK, JNK, and p38 MAPK, as well as TAK1.

Besides Smurf1, several E3 ubiquitin ligases are involved in the regulation of the TGF-β pathway. For instance, TβRI is reported to be polyubiquitinated by WW domain-containing protein 1 (WWP1) and neural precursor cell expressed developmentally downregulated (NEDD)-4, which alter both receptor stability on the membrane and internalization, thus restricting cell sensitivity toward TGF-β stimulation [39,40]. SMAD2 can be polyubiquitinated by Smurf2 and NEDD-4 [40,41], SMAD3 by C-terminus of HSC70-interacting protein (CHIP) [42], and SMAD4 by either Smurf1/2, WWP1 or NEDD-2[43]. Additionally, the nuclear RING-domain E3 ligase Arkadia has been reported to ubiquitinate the phosphorylated SMAD2/3, causing their degradation and preventing their nuclear accumulation [44]. The deubiquitinating enzyme USP26 limits the ubiquitin-mediated turnover of SMAD7, increasing its stability [45].

Adaptor proteins such as the SMAD anchor for receptor activation (SARA), embryonic liver fodrin (ELF), and microtubules have also been involved in the proper control of SMAD’s access to the TβRI at the cell membrane, which is required for their activation and nuclear translocation [46,47].

## 4. TGF-β Signaling in Cancer

During the early stage of tumorigenesis, TGF-β operates as a tumor suppressor by inducing apoptosis in pre-malignant cells or G1 phase cell cycle arrest in carcinoma cells [48]. Previous studies have implicated p15^INK2B^ and p27^Kip1^ as mediators of the growth inhibitory effect of TGF-β, which act by inhibiting cyclinD1/Cdk4 and cyclinE/Cdk2 complexes, respectively [49,50]. Further, TGF-β has been shown to induce p21 (Waf1, Cip1) through a p53-independent transcriptional regulation [51,52,53] but also by increasing its protein stability, which leads to inhibition of Cdk2 kinase activity [54].

At later stages, when tumor cells have acquired different oncogenic mutations and lost tumor suppressor gene function, they become resistant to the cytostatic effect of TGF-β and reinterpret TGF-β signals to favor the so-called epithelial-to-mesenchymal transition (EMT) [10,55]. EMT is a biological process that endows tumor cells with a mesenchymal phenotype characterized by downregulation of E-cadherin (epithelial marker), gain of N-cadherin and vimentin (mesenchymal markers), and increased secretion of matrix metalloproteinases (MMPs) [56]. These changes lead to decreased cell–cell and cell–matrix connections, loss of cell polarity, and increased interactions between tumor and stromal cells, favoring the initial phase of metastatic dissemination [57]. TGF-β-activated SMADs regulate the transcription of EMT-related genes, including Snail, Zinc finger E-Box-Binding Homeobox (ZEB), and basic/Helix-Loop-Helix (bHLH), which can suppress the expression of epithelial markers and induce the expression of mesenchymal ones [58,59].

TGF-β also signals in a paracrine manner to promote tumor progression, interfering with the cell–cell connections that occur in the tumor microenvironment (TME). TGF-β may alter the ECM composition to induce a fibrotic and inflammatory environment enriched in growth factors, matrix proteins, and proteases, and can also modulate the physical properties of EMC, such as tension and stiffness, thus impacting the invasive abilities of cancer cells and promoting metastatic dissemination [60,61,62]. Also, the pro-angiogenic function of TGF-β signaling relies on its ability to induce the expression of key angiogenic factors such as vascular endothelial growth factor (VEGF), connective tissue growth factor (CTGF), and insulin-like growth factor-binding protein 7 (IGFBP7) by tumor cells [63,64,65], but also to stimulate ECM production by fibroblasts and to drive tube formation by endothelial cells [66]. TGF-β has also been shown to drive changes in both stromal and immune cells of TME to induce a strong immune-suppressive milieu [67].

## 5. TGF- in Gastrointestinal Tumors

TGF-β has dual roles in different stages of GI tumor initiation and progression (summarized in Table 1). The acquisition of loss-of-function mutations in core elements of the TGF-β pathway such as SMAD proteins and TGF-β receptors has been shown to contribute to the functional switch of TGF-β from tumor-suppressive to tumor-promoting, allowing tumor cells to grow in a TGF-β-enriched microenvironment [68]. The interconnections between TGF-β and signaling pathways that are differentially activated during progression and metastasis may also contribute to this functional switch, as multiple co-activators and co-repressors of SMAD transcription factors could be responsible for context- and time-dependent transcriptional regulation of different downstream effectors [69].

### 5.1. Pancreatic Cancer

Pancreatic cancer (PC) is a highly aggressive tumor, with a 5-year survival rate of less than 6% and median survival from diagnosis of about 6 months. PC is characterized by increasing incidence, rapid progression, and resistance to radio- and chemotherapy [101,102]. It is usually diagnosed at advanced stages, and it is expected to become the second leading cause of cancer-related death worldwide by 2030 [103]. Pancreatic ductal adenocarcinoma (PDAC) represents the major histological subtype, comprising 90% of all PC cases [104].

Loss of SMAD4 occurs in about 50–60% of PC cases, either by loss of heterozygosity at 18q21 or by inactivating mutations, and correlates with poor prognosis in PC patients [105]. Inactivating mutations in SMAD4 have been reported to accelerate the malignant transformation of pancreatic duct cells initiated by activated KRAS [106,107,108]. Inactivation of SMAD4 is often a late event in the development of pancreatic adenocarcinoma, occurring after exceeding the normal growth constraints, and has been related to increased metastatic potential [72,73].

PC tissues frequently show increased circulating levels of TGF-β isoforms [109], which correlate with decreased survival [74,110] and higher incidence of distant metastasis [74]. Enhanced expression of TGF-β ligands has been associated with increased EMT and invasion, stimulation of fibrosis, angiogenesis, and immune suppression [3,4,5,76]. This may occur via different mechanisms. For instance, Zhang et al. demonstrated that although TGF-β mediates activation of SMAD3 in pancreatic carcinoma cells with subsequent cell growth arrest, increased TGF-β can also activate SMAD7, which induces nuclear translocation of β-catenin and promotes vascularization and metastasis through the induction of VEGF-A [75]. Furthermore, Cheng et al. reported that increased TGF-β ligand in PC cells downregulates CCAAT/enhancer-binding protein (C/EBP) beta (C/EBPβ), with subsequent downregulation of the epithelial genes *CDH1* and *CLDN3*, and induction of the EMT program. The authors propose that a SMAD3/Menin complex could interact with the HDAC complex to promote C/EBPβ repression, functioning as an oncogenic complex that drives EMT and cancer metastasis [70].

The extensive crosstalk between SMAD proteins and other signaling pathways is also critical for PC progression. Wang et al. showed that the oncogene Methyl-CpG-binding protein 2 (MeCP2) activates the TGF-β1 ligand, leading to increased phosphorylation of SMAD2/3 without interfering with the expression of TGF-β receptors. MeCP2 functions as a transcriptional co-activator of SMAD3 in mediating the SMAD-dependent transcriptional activation of Furin, which is a member of the family of subtilisin/kexin-like proprotein convertases that has been shown to promote migration and invasion of tumor cells, forming a positive feedback axis that promotes EMT in PC cells [71]. In another study, Otsuru et al. reported that the inflammation-related molecule leucine-rich alpha-2 glycoprotein (LRG) increases the expression of TGFBR1 and enhances the TGF-β1-induced SMAD phosphorylation in PDAC patients, potentiating the EMT induced by SMAD2 signals [111]. Non-canonical SMAD signaling is also crucial in the tumor-promoting functions of TGF-β. The TGF-β-activated kinase 1 (TAK1), a protein kinase essential in the activation of Nuclear Factor-κB (NF-κB), is a downstream target of TGF-β and has been reported to reduce pro-apoptotic pathways and enhance chemoresistance in PC [77,78]. As proof, reducing TAK1 protein stabilization through the inhibition of the serine/threonine kinase Glycogen Synthase Kinase-3alpha (GSK-3alpha) has been shown to prolong the survival of mice treated with the compound nab-paclitaxel [78]. It was recently demonstrated that the TAK1-regulated circulating factor IL-8 functions as a predictive biomarker of resistance to the nano-liposomal irinotecan in gemcitabine-refractory PC patients [112].

### 5.2. Colorectal Cancer

Colorectal cancer (CRC) represents the third leading cause of cancer worldwide and accounts for about 8–9% of cancer-related deaths [113]. CRC mortality rate is declining in Western countries due to increased prevention, earlier detection, and improved treatment options. However, patients with late-stage CRC acquire resistance to adjuvant therapy and still die from metastatic dissemination [114]. Depending on the genetic expression, CRC can be classified into four different consensus molecular subtypes (CMSs): *CMS1*, which is associated with immune infiltration; *CM2S* (classical CRC), which is characterized by chromosomal instability, epithelial signature, and activation of WNT pathway; *CMS3*, which presents a strong epithelial composition, modification of different processes associated with metabolism and *KRAS* mutations; and *CMS4*, which shows a more mesenchymal signature, activation of EMT-related genes, remodeling of extracellular matrix, and activation of TGF-β signaling [115]. De Sousa et al. demonstrated that relapse in CRC patients mainly occurs in the CMS4 subtype [116].

In the normal intestinal epithelium, TGF-β provides growth-inhibitory signals that are restrained during CRC progression by inactivating mutations in components of the TGF-β pathway. Mutations in TβRII is the most common mechanism of loss of TGF-β signaling in CRC [117,118,119], and inactivating mutations in this gene contribute to the malignant phenotype when combined with the activation of multiple signaling pathways such as Wnt-β-catenin, MAPK, or Hippo pathways [120]. Patients with Lynch syndrome rapidly develop CRC due to inactivation of DNA mismatch repair (MMR) genes (MLH1, MSH2, MSH6, or PMS2) that follow germline mutations or MMR promoter hypermethylation [121,122]. This event has been shown to promote microsatellite instability in several genes, including TGFBR2 [117,123]. The effect of TGFBR2 loss in colon cancer formation was investigated in a conditional knockout Cre-Tgfbr2(flx/flx) mouse model, in which genetic ablation of the Tgfbr2 gene led to increased proliferation due to the inability to inactivate Cdk4 expression and kinase activity [81]. Inactivating mutations in SMAD2, SMAD3, and SMAD4 genes have also been observed in sporadic CRC [79,124,125] and have been suggested to reduce their protein stability or to interfere with the formation of SMAD complexes involved in downstream transcriptional responses [124]. Clinically, decreased expression of SMAD4 has been related to shorter patient survival [126]. Studies in mouse models have shown that homozygous loss of SMAD4 led to the transformation of intestinal polyps into malignant tumors only in the context of a primed, APC-defective genetic background [80]. Further, simultaneous loss of SMAD4 and Wnt pathway activation led to de-differentiation and adenoma formation in the differentiated intestinal epithelium of the Cre-driven conditional mouse model [80,127]. Zhang et al. suggested a role for SMAD4 in mediating the functional switching of TGF-β from tumor suppressor to tumor promoter. The authors demonstrated that genetic depletion of SMAD4 in colon cancer cells allows TGF-β-induced proliferation, migration, and invasion, whereas its ectopic re-expression reverts the tumorigenic effects induced by TGF-β [82].

Increasing evidence highlight the role of non-canonical TGF-β signaling pathways in mediating CRC metastasis in a SMAD4-defective context. Although the loss of SMAD4 inhibits the canonical TGF-β signaling, it has also been shown to induce BMP signaling to switch from tumor suppression to increased EMT, invasion, and metastasis promotion, which occur via the activation of Rho signaling through Rho-associated protein kinase (ROCK) and LIM domain kinase (LIMK) [85]. Loss of SMAD4 has also been shown to induce alternative ERK pathways to induce migration and invasion of colon cancer cells in vitro, to enhance liver metastasis in vivo, and to shorten the survival of metastatic tumor-bearing mice [84]. An intriguing work by Gatza and colleagues reported that overexpression of TβRIII betaglycan in colon cancer cells enhances both canonical and non-canonical TGF-β signaling by inducing TGF-β-dependent phosphorylation of SMAD2 and p38 while inhibiting TGF-β -induced p21 and p27 expression. This turns off the TGF-β pro-apoptotic signal on behalf of sustained proliferation and increased migration [83].

### 5.3. Gastric Cancer

Gastric cancer (GC) is the second leading cause of cancer-related death worldwide. Both environmental and genetic factors have a role in its etiology [128]. Despite early diagnosis and treatment with a combination of surgery, chemotherapy, and/or radiotherapy [129], the prognosis of GC patients remains poor due to relapse and distant metastasis [130]. Previous studies have shown increased serum levels of TGF-β in GC patients compared to healthy controls, which have been correlated with decreased overall survival, worse prognosis, and lymph node metastasis [86,131]. Mutations in TGFBR2 have also been associated with gastric carcinogenesis [132], while epigenetic silencing of TGFBR1 has been correlated with poorer prognosis in GC patients [133].

Additional regulatory mechanisms may also contribute to the activation of TGF-β signaling. Aberrantly expressed MDS1/EVI 1 like gene 1 (MEL1) in GC cell lines has been shown to interact with the SMAD co-repressor SKI in inducing the stabilization of the inactive SMAD3-SKI complex, leading to the inhibition of the TGF-β signaling pathway [90]. Consistently, depletion of both MEL1 and SKI restores TGF-β sensitivity in GC cells leading to reduced tumor growth in vivo [90]. Li et al. identified Runx-related transcription factor 3 (RUNX3) as a tumor suppressor co-factor for SMAD proteins, potentiating their interaction with target DNA [91]. Loss of RUNX3 due to promoter hypermethylation is frequently observed in GC [91], and mutation of RUNX3 at (R122C) has also been reported to abolish its ability to cooperate with SMAD proteins in inducing p21 expression in epithelial cells of the stomach [134]. Zhang et al. reported that TGF-β1 is a direct transcriptional target of Spalt Like Transcription Factor 4 (SALL4), a zinc-finger transcription factor that is involved in pluripotency and renewal of embryonic stem cells. [89]. The authors report a positive correlation between TGF-β1 and SALL4 in GC tissues and provide evidence that TGF-β1 acts as a downstream effector of SALL4 in GC progression, as its knockdown reverses SALL4-mediated promotion of GC migration, invasion, and metastasis [89]. Xiong and colleagues found that GTPase-Activating Protein SH3 Domain-Binding Protein 1 (G3BP1), which is higher expressed in GC correlating with poor prognosis and metastasis, controls the activation of the TGF-β/SMAD pathway in GC cells by directly regulating the expression of TGF-β1 and TGF-β2 ligands, thus altering the phosphorylation of SMAD2/3 [87].

Although the role of TGF-β has been not yet fully elucidated in GC, a compensatory balance between SMAD4 and SMAD7 proteins has been suggested to allow the functional switch of TGF-β toward de-differentiation and lymphatic metastasis in GC [88]. Additionally, signals from SMAD-dependent and SMAD-independent pathways may cooperate during TGF-β-mediated GC progression. In support of that, TGF-β1 has been shown to induce angiogenesis in GC cells by increasing the expression of *VEGFC* both canonically and via the SMAD-independent AKT pathway [64].

### 5.4. Hepatocellular Carcinoma

Liver cancer is one of the leading causes of global cancer mortality [135]. Primary liver cancers mainly include hepatocellular carcinoma (HCC) (75–85% of cases) and intrahepatic cholangiocarcinoma (10–15% of cases) [136]. HCC often develops in a cirrhotic microenvironment caused by viral infections, steatohepatitis, alcoholism, or metabolic disorders, in which persistent liver inflammation causes the accumulation of excessive EMC, the substitution of parenchymal cells with fibrotic tissue, and impairment of the liver function, finally leading to the development of HCC [137,138]. TGF- β1 plays a critical role in this process. In normal liver, it induces apoptosis of hepatic cells, for instance, by inducing activation of p38 by growth arrest and DNA damage-inducible protein (GADD45)-β [139], or of JNK by the adapter protein Death-associated protein 6 (Daxx) [140]. When the tumor progresses, TGF-β1 stimulates the expression of several EMC proteins and integrins, cellular receptors for ECM proteins, whose activation alters the expression and/or function of TGF-β downstream effectors, allowing the escape of hepatocytes from apoptosis induced by TGF-β1 [141]. For instance, Zhang and colleagues reported that overexpression of β1-integrin has been shown to overcome TGF-β1-induced apoptosis [142]. The authors showed that overexpression of β1 integrin in HCC cells induces the activation of the MAPK pathway and that TGF-β1 further induces sustained activation of JNK, p38, and ERK signaling pathways. Inhibition of these pathways reverses the apoptosis of hepatocytes induced by TGF-β1, thus suggesting a critical role for the MAPK signaling pathway in the escape of HCC cells from TGF-β-induced apoptosis [142].

A large body of evidence also supports the role of TGF-β signaling in HCC progression. Wang et al. showed that the function of the Signal Transducer and Activator of Transcription 3 (STAT3) positively regulates the TGF-β1 mediated EMT [92]. Mazzocca and collaborators reported that pharmacological inhibition of the TGF-β signaling pathway with the TβRI kinase inhibitor LY2109761 impairs tumor angiogenesis by both inhibiting SMAD-dependent VEGF production in HCC cells and hampering the crosstalk between tumor cells and microenvironment, thus inhibiting migration of endothelial cells [94]. Bhagyaraj et al. highlighted a key role for TGF-β signaling in the onset of chemoresistance, which occurs through the TGF-β-induced upregulation of xenobiotic nuclear receptor (PXR), which in turn enhances the expression of drug-efflux transporters [95].

Several regulatory mechanisms have been involved in the modulation of TGF-β signaling depending on the cellular context and stage. For instance, Yan et al. identified the CXXC-type zinc finger protein 5 (CXXC5) as a positive regulator of TGF-β signaling. The authors demonstrated that CXXC5 acts as a tumor suppressor by associating with the histone deacetylase HDAC1 and competing with it for the interaction with SMAD2/3 protein complex, thus abolishing the inhibitory effect of the deacetylase on TGF-β signaling and leading to apoptosis and growth inhibition [96]. Liu and colleagues showed that the PSMD14 protein POH1 hyperactivates TGF-β signaling and promotes HCC metastasis by deubiquitinating TGF-β receptors, thus reducing their lysosome-dependent turnover [97]. Recently, Yang et al. demonstrated that the Golgi protein 73 (GP73) promotes EMT and invasion in HCC cells by inducing the activation of SMAD2 and SMAD3 through phosphorylation [93]. The adaptor protein embryonic liver fodrin (ELF) has also been involved in the modulation of TGF-β functions. The authors found that TGF-β triggers phosphorylation of ELF, which subsequently associates with SMAD3 and SMAD4 and facilitates their accumulation in the nucleus. Furthermore, Elf ^-/-^ mice induce spontaneous development of HCC [47].

The crosstalk between TGF-β, growth factors, and integrins has also been reported to alter the phosphorylation of SMAD proteins at their C-terminus and linker region, contributing to the switch of TGF-β signals from tumor suppression to tumor promotion. Indeed, TGF-β exerts its growth-inhibitory effects through the TβRI-mediated SMAD2/3 phosphorylation at their C-terminus, within the SSXS motif. However, activation of different kinases such as ERK, p38, JNK, MAPK, CDKs, and ROCK, has been shown to induce the phosphorylation of SMAD2 and SMAD3 in their linker regions, which results in altered transcriptional activity of the SMAD2/3 complex and the functional switch from apoptosis to EMT and tumor progression [143,144].

### 5.5. Esophageal Cancer

Esophageal cancer (EC) represents the eighth most common tumor, with esophageal squamous cell cancer (ESCC) and esophageal adenocarcinoma that cover more than 95% of tumor cases [145].

In ESCC, the X-linked inhibitory of apoptosis protein (XIAP) has been reported to positively modulate the ability of tumor cells to undergo TGF-β-dependent EMT, allowing tumor cell migration [98]. Liu et al. identified a positive correlation between TGF-β1 and Metastasis-Associated Lung Adenocarcinoma Transcript 1 (MALAT1), which cooperate in inducing the upregulation of the mesenchymal markers and the downregulation of the epithelial ones [99]. Wang and colleagues showed that phosphorylated and active SMAD2/3 interact with E1A-associated binding protein p300 in the nucleus and that this interaction is required to increase the acetyltransferase activity of p300, leading to increased cell proliferation and migratory ability [100]. The expression of p300 has been shown to correlate with poor prognosis in ESCC patients [146]. Thus, inhibition of TGF-β signaling represents a promising therapeutic strategy for ESCC. For instance, the natural compound Garcinol, which acts as a histone acetyltransferase, has been shown to decrease phosphorylation levels of SMAD2/3, inhibiting ESCC metastasis both in vitro and in vivo [100].

## 6. TGF-β and Tumor Microenvironment

During late-stage cancer, sustained release of TGF-β by tumor cells alters the TME to allow tumor growth, invasion, and metastasis [147,148]. This is achieved by directly suppressing immunosurveillance by inhibiting the anti-tumor activity of infiltrating immune cells while facilitating the recruitment and sustaining the function of pro-tumoral immune cells that enhance metastatic progression. The extensive role of the TGF-β signaling pathway in regulating immune cell functions has been reviewed in detail elsewhere [149,150,151,152]. Here, we provide an overview of the main activities of TGF-β on macrophages, neutrophils, myeloid-derived suppressor cells (MDSCs), T cells, and cancer-associated fibroblasts that enhance tumor development and progression with some evidence for GI tumors. The contribution of the crosstalk between tumor cells and TAMs in GI tumor development and immune evasion has been reviewed in detail elsewhere [149,150,151,152].

### 6.1. Regulation of Macrophage Plasticity

Macrophages can be differentiated into subsets with distinctive phenotypes. Classically activated M1 macrophages are pro-inflammatory and exhibit anti-tumor activity, while alternatively activated M2 macrophages are anti-inflammatory and exhibit pro-tumor activity. Tumor-associated macrophages (TAMs), which represent one of the most abundant immune cell populations in the TME, resemble M2 macrophages [153].

TGF-β induces specific programs in monocytes/macrophages lineage depending on context and differentiation state. Previous studies reported that TGF-β recruits monocytes to the site of inflammation [154,155] and that their differentiation into perivascular macrophages favors cancer cell intravasation [156]. TGF-β secreted by tumor cells has also been shown to induce M2-type polarization of macrophages, which acquire immune-suppressive, anti-inflammatory, and pro-angiogenic functions [157]. Standiford et al. reported that tumor-derived TGF-β induces polarization of macrophages toward M2 by increasing the expression of interleukin-1 receptor-associated kinase-M (IRAK-M), which is a negative regulator of Toll-like receptor (TLR) signaling [158]. Zhang and colleagues found that TGF-β can also favor M2 polarization via Snail upregulation, which occurs via SMAD2/3 and PI3K/AKT signaling pathway [159]. Other evidence showed that TGF-β1 can suppress the inflammatory M1-phenotype of macrophages by crosstalk with TNF signaling pathway through SMAD7, which blocks the activity of TAK1 [160].

TGF-β is also one of the main immunosuppressive cytokines produced by TAMs [161]. In CRC recruited TAMs produce TGF-β ligand to promote EMT and invasion via VEGF [162,163]. In PDAC, TAMs enhance cancer cell migration by inducing EMT through the TGF-β-SMAD2/3/4-Snail axis [164]. Although these mechanisms have not been yet fully explored in the context of GI tumors, it is reasonable that a TGF-β-rich TME could contribute to immune evasion turning off the inflammatory function of macrophages.

### 6.2. Regulation of Neutrophil Plasticity

Neutrophils represent about 70% of leukocytes and function in the healing of damaged tissues and in resolving infections. Increased numbers of circulating neutrophils in patients with breast, lung, and colon cancers have been often associated with poor prognosis [165]. TGF-β acts as a potent chemoattractant for neutrophils [166], and inhibition of TGF-β signaling has been shown to enforce the anti-tumoral phenotype in tumor-associated neutrophils (TANs) that includes cytolytic activity and increased expression of pro-inflammatory cytokines [167].

Chronic inflammation and cancer induce the persistent production of cells like neutrophils and monocytes, which are endowed with the ability to suppress immune responses. These MDSCs are able to infiltrate tumors, functioning as important players during cancer immune evasion. [168]. The contribution of MDSCs to TGF-β-induced EMT has only recently started to emerge. In mouse models, a population of CD11b+Gr1+ cells were shown to infiltrate primary tumors in a mechanism that rely on TGF-β, and that their depletion resulted in smaller tumors coupled with a severe reduction in metastasis. In vitro experiments have shown that TGF-β mediates this effect [169]. Further, depletion of MDSCs in a breast cancer model has been shown to inhibit the therapeutic effects exerted by systemic administration of anti-TGF-β antibodies [170].

### 6.3. Induction of T Regulatory Phenotype

Infiltrating T cells are activated through signals to the T-cell receptor (TCR) from a complex of major histocompatibility complex (MHC) and antigenic peptide, and to the T cell surface receptor CD28 by the costimulatory molecules CD80 or CD86 on antigen-presenting cells (APC). Once activated, the expression of co-inhibitory receptors such as cytotoxic T lymphocyte antigen 4 (CTLA4) and programmed cell death 1 (PD-1) increases in T cells, which become exhausted T cells [171,172]. In CRC, TGF-β promotes T cell exclusion of both CD4+ and CD8+ cells from the tumor mass and blocks the acquisition of a Th1-effector phenotype [173]. TGF-β has also been shown to suppress the expression of effector function on memory CD8+ T cells and reactive tumor-infiltrating lymphocytes, likely hindering the benefits of immunotherapy in cancer patients [174]. Resident memory T cells (Trm) represent a subset of CD8+ T cells mainly found in the mucosa, in contact with the surrounding microenvironment of the digestive tract and lung and are characterized by the inability to recirculate [175]. TGF-β has been shown to act as a crucial player in Trm formation and maintenance by inducing the expression of the integrin CD103 [176]. Consistently, inhibition of TGF-β with the small molecule Galunisertib induced a potent cytotoxic T cell response against CRC cells, keeping tumors more susceptible to anti-PD-L1/PD-1 therapy [173].

Several lines of evidence pointed to the involvement of TGF-β in promoting phenotypical changes of T cells to regulatory T cells (Treg), which lead to tumor progression [177]. Indeed, T-regs are reported to contribute to establishing an immune-suppressive TME, which promotes cancer progression [178]. For instance, tumor-secreted TGF-β favors the polarization of CD4+ T cells in Treg cells in PC mouse models [179]. Further, it is responsible for the transcriptional activation of Foxp3, which induces the CD4+CD25+ phenotype in Treg, with subsequent suppression of CD8+ T cell cytotoxic activity [180,181,182,183], and also for mediating the polarization of Th17 cells from naïve T cells [184] by suppressing IL-23R and favoring Foxp3+ Treg, which in turn inhibit the function of RAR-related orphan receptor gamma (RORgt) [185]. TGF-β1 and IL-6 have been reported to mediate the release of IL-17 from Th17 cells, strongly increasing the inflammatory response [186]. In biliary tract cancers, differential secretion of TGF-β1 and IL-6 is one of the main causes of the heterogeneous distribution of Treg and Th17 cells in the TME, with the tumor center enriched in the Treg subpopulation, and the tumor invasion front mainly represented by Th17 cells [187]. Another important example is provided by Wang et al., in which the authors demonstrated that PC patients display a Th17/Treg ratio that outlines an increment of the amount Tregs at the expense of the number of Th17. This unbalanced proportion leads to an aberrant cytokines production that plays a central role in the development and tumorigenesis of PC [188].

### 6.4. Crosstalk with Cancer Associated Fibroblasts

Cancer-associated fibroblasts (CAFs) represent one of the main stromal cell types in the tumor microenvironment. CAFs secrete and remodel the extracellular matrix [189] and favor malignant progression by endowing tumor cells with proliferative, migratory, and invasive abilities [190]. CAFs can derive from fibroblasts that are already present in the tumor microenvironment, from bone marrow mesenchymal stem cells, or from cancer epithelial and endothelial cells [191,192,193,194,195]. TGF-β is one of the most potent mediators of the differentiation process responsible for the polarization into CAFs [196].

In GI stromal tumors, TGF-β has been reported to mediate the trans-differentiation of resident fibroblasts into CAFs acting in a paracrine manner [197]. In PC, TGF-β has been shown to antagonize IL-1R1, which is responsible for switching CAF to an inflammatory subtype (iCAF), leading to fibroblast polarization into a myofibroblastic phenotype (myCAF) [198] that is characterized by increased expression of alpha-smooth muscle actin (αSMA) and fibroblast activation protein (FAP) [199]. In CRC patients, TGF-β has been shown to activate a gene expression program in CAFs that is associated with a worse prognosis in CRC patients [200]. Hyperactivation of TGF-β signaling in CAFs has been shown to contribute to the invasive abilities of cancer cells and metastatic dissemination [200,201,202]. Additionally, the TGF-β family co-receptor endoglin has been involved in CAF-mediated invasion and metastasis into the liver in CRC [203]. CAFs represent another major source of TGF-β in several tumors. Paracrine TGF-β1 signaling by CAFs has been reported to promote EMT in cancer cells [204].

## 7. TGF-β Targeted Therapies

Different strategies have been developed to interfere with the TGF-β signaling pathway, some of which are currently explored in clinical trials. These include agents that interfere with TGF-β synthesis, with ligand-receptor interaction, or with receptor kinase activity.

The first group includes antisense oligonucleotides (ASOs), which are synthetic short single-stranded RNAs designed to inhibit the translation of TGF-β mRNA [205,206,207] or to induce exon skipping of the type I TGF-β receptor ALK5 [208]. Based on the observed efficacy in preclinical studies, ASOs have been used in several clinical trials [209,210]. For instance, AP12009 (Trabedersen), which targets TGF-β2 mRNA, has been successfully used to treat melanomas, colon, and pancreatic cancers in a phase I study (NCT00844064) Schlingensiepen et al. demonstrated that Trabedersen decreases TGF-β2 levels in PC cells, inhibiting cell proliferation and migration. Tumor growth as well as angiogenesis and lymph node metastasis were also strongly reduced in orthotopic xenograft mouse models of PC treated with AP12009 [211].

The second group includes monoclonal antibodies that are designed to disrupt the interactions between TGF-β ligands and their receptor [212]. These compounds can hamper the availability of TGF-β and reduce SMAD2/3 phosphorylation as well as the expression of TGF-β target genes [213]. As an example, the pan-TGF-β neutralizing antibody 1D11 blocks TGF-β-induced phosphorylation of receptor-associated SMADs, inhibiting TGF-β-mediated migration and invasiveness of breast cancer cells [214]. Similarly, the pan-specific TGF-β monoclonal antibody GC1008, also known as Fresolimumab, has been shown to bind to all three TGF-β isoforms, reducing their binding to the receptor [215,216]. To date, Fresolimumab has been used in phase I/II studies in various types of cancer, whilst it has not been yet investigated on GI tumors. A phase I study with the anti-TGFβR2 monoclonal antibody LY3022859 in patients with advanced solid tumors (NCT01646203) failed in determining the maximum tolerated dose (MTD), as dose escalation beyond 25 mg was related to worsening symptoms, such as uncontrolled cytokine release [217].

The third group is represented by kinase inhibitors. These compounds reduce the kinase activity of type I and type II TGF-β receptors by competing with ATP binding and have been shown to reduce both tumor growth and metastasis in preclinical studies [218,219,220]. For instance, the TβRI inhibitor Ki26894, known as Kirin, showed inhibitory effects in GC tumors by reducing invasiveness and bone metastasis development [220]. The oral inhibitor of the TβRI kinase activity Vactosertib has been shown to suppress tumor progression in mouse models by acting through both intrinsic and extrinsic mechanisms [221,222]. Clinical trials in solid tumors (NCT02160106) have also confirmed the efficacy and safety of Vactosertib in several tumor types. Another small molecule that inhibits the kinase activity of TβRI is LY2157299, also known as Galunisertib. Its efficacy has been demonstrated in a phase 1b/2 study in patients with unresectable pancreatic cancer when in combination with gemcitabine, with minimal added toxicity [223,224]. Safety, efficacy, and pharmacokinetics of Galunisertib in combination with the anti-PD-1 antibody Nivolumab (NCT02423343) or with anti-PD-L1 antibody Durvalumab (NCT02734160) [225] have also been demonstrated recently in phase I/II trials. In HCC patients that were ineligible for Sorafenib, treatment with Galunisertib has been reported to increase the overall survival (7.91 months in patients with a reduction of TGF-β less than 20% vs. 21.8 months in patients with a reduction of TGF-β more than 20%) [226]. Increased apoptosis and reduced tumor growth were also achieved by combining Galunisertib and Sorafenib in HCC patients [227].

Altogether, these data highlight the importance of targeting of TGF-β pathway to improve the overall survival and progression-free survival in patients with advanced and refractory tumors. However, although these strategies have been proven successful in both in vitro and in vivo models, results from clinical trials frequently show only minimal survival benefits and occasional adverse effects, such as cardiovascular toxic side effects or benign tumor formation. This could rely on different aspects. First, the inappropriate selection of patients for clinical trials could mask the efficacy of the treatment. Second, the schedule of treatment administration in mouse models begins at an early stage when a tumor is palpable. Differently from animal models, patients could not be diagnosed until the symptoms’ appearance, which frequently occurs at later stages, when the EMT process has been completed, and tumor cells have already spread and metastasized. Third, the dynamics of TGF-β signaling during tumor progression are under the control of negative feedback loops and crosstalk mechanisms with other signaling pathways. Furthermore, as the effects of TGF-β inhibitors rely on inhibition of cancer invasion and metastasis, it is of critical importance to combine anti-TGF-β therapies with cytostatic drugs to counteract tumor growth [209,228] or with immune checkpoint inhibitors that can counteract the immune suppressive effects of TGF-β.

## 8. Conclusions

**Figure 2:**
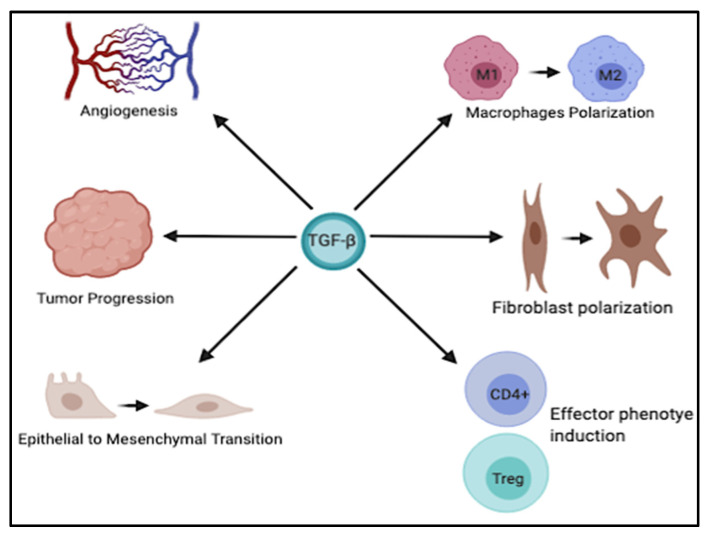
Schematic representation of the multifaceted role of TGF-β. TGF-β is implicated in various physiological and pathological processes such as angiogenesis, tumor progression, EMT, and polarization of different immune and stromal cells. This figure was created with BioRender.com, accessed on 4 June 2021.

It is well known that dysregulation of TGF-β signaling is closely associated with the development of various GI tumors. The multifaceted role of TGF-β is exploited by the ability of this cytokine to modulate different biological processes, including EMT, angiogenesis, immune cells plasticity, and late stages of tumor progression (Figure 2). However, a better understanding of the molecular mechanisms underlying its dichotomous role in the progression of these cancers, as well as its intricate crosstalk with other signaling pathways, is of critical importance to develop more effective therapeutic strategies, perhaps in combination with other agents to boost the therapeutic benefits and improve the clinical outcome in these tumors.

## Figures and Tables

**Table 1:** Summary of the main roles of the TGF-β signaling pathway in GI tumors that are described in this review.

	Role of TGF-β signaling in GI Tumors
Pancreatic Cancer	- Epithelial-mesenchymal transition [4,70,71] - Migration [71] - Invasion [71] - Metastasis [70,71,72,73,74,75] - Fibrosis [76] - Angiogenesis [75] - Chemoresistance [77,78]
Colorectal Cancer	- Malignant phenotype promotion [79,80] - Evading growth suppression [81] - Migration [82,83,84] - Invasion [82,84,85] - Metastasis [84,85]
Gastric Cancer	- Metastasis [86,87,88,89] - Tumor growth suppression [90,91] - Migration [89] - Invasion [89] - Angiogenesis [64]
Hepatocellular Carcinoma	- Epithelial–mesenchymal transition [92,93] - Migration [92,93] - Angiogenesis [94] - Chemoresistance [95] - Apoptosis and growth suppression [96] - Metastasis [97] - Invasion [93]
Esophageal Cancer	- Epithelial–mesenchymal transition and migration [98,99] - Migration [98,99] - Invasion [99] - Metastasis [100]

## Abbreviation

α-SMA	a-Smooth Muscle Actin
ALK1–7	Activin Receptor-Like Kinase 1–7
APC	Antigen-Presenting Cells
ASO	Antisense Oligonucleotides
bHLH	Basic/Helix-Loop-Helix
BMPs	Bone Morphogenetic Proteins
C/EBPb	CCAAT/Enhancer-Binding Protein beta
CAFs	Cancer-Associated Fibroblasts
CDK	Cyclin-Dependent Kinase
CMSs	Consensus Molecular Subtypes
CRC	Colorectal Cancer
CTGF	Connective Tissue Growth Factor
CTLA4	Cytotoxic T Lymphocyte Antigen 4
CXXC5	CXXC-type zinc finger protein 5
DAXX	Death-Associated protein 6
EC	Esophageal Cancer
ECM	Extracellular Matrix
ELF	Embryonic Liver Fodrin
EMT	Epithelial to Mesenchymal Transition
ESCC	Esophageal Squamous Cell Carcinoma
ERK	Extracellular Signal-Regulated Kinase
FAP	Fibroblast Activation Protein
G3BP1	GTPase-Activating Protein SH3 Domain-Binding Protein 1
GC	Gastric Cancer
GI	Gastrointestinal
GP73	Golgi Protein 73
GSK-3alpha	Glycogen Synthase Kinase-3alpha
HCC	Hepatocellular Carcinoma
HDAC	Histone Deacetylase
iCAF	Inflammatory CAF
IGFBP7	Insulin-like Growth Factor-Binding Protein 7
IRAK-M	Interleukin-1 Receptor Associated Kinase-M
JNK	c-Jun N-terminal Kinases
LAP	Latency-Associated Peptide
LIMK	LIM domain Kinase
LLP	Large Latent Complex
LRG	Leucine-Rich alpha-2 Glycoprotein
LTBP	Latent TGF-β Binding Protein
MALAT1	Metastasis-Associated Lung Adenocarcinoma Transcript 1
MAPK	Mitogen-Activated Protein Kinases
MTD	Maximum Tolerated Dose
MDSCs	Myeloid-Derived Suppressor Cells
MeCP2	Methyl-CpG-Binding Protein 2
MEL1	MDS1/EVI 1 Like gene
MHC	Major Histocompatibility Complex
MMPs	Matrix Metalloproteinases
MMR	Mismatch Repair
myCAF	Myofibroblastic CAF
NF-κB	Nuclear Factor-κB
PC	Pancreatic Cancer
PD-1	Programmed Cell Death 1
PDACs	Pancreatic Ductal Adenocarcinomas
PI3K	Phosphoinositide 3-Kinase
PXR	Xenobiotic Nuclear Receptor
ROCK	Rho-associated Protein Kinase
R-SMAD	Receptor Regulated SMAD
RGD	Arginine-Glycine Aspartate
RHO	Rhodopsin
RORGT	RAR-related Orphan Receptor Gamma
RUNX3	Runx-related Transcription Factor 3
SALL4	Spalt Like Transcription Factor 4
SLC	Small Latent Complex
SMAD	Acronym obtained by merging *Caenorhabditis elegans Sma* genes and the *Drosophila Mad*, Mothers against decapentaplegic
SMURF	SMAD Ubiquitination Regulatory Factor
STAT3	Signal Transducer and Activator of Transcription 3
TAK1	TGF-β-activated kinase-1
TAMs	Tumor-Associated Macrophages
TANs	Tumor-Associated Neutrophils
TβRI-III	Type I-III Receptors
TCR	T-cell Receptor
TGF-βR	Transforming growth factor-beta receptor
TGF-β	Transforming growth factor-beta
TLR	Toll-like Receptor
TME	Tumor Microenvironment
TREG	T Cells to Regulatory T cell
TRM	Resident Memory T cell
VEGF	Vascular Endothelial Growth Factor
VEGFC	Vascular Endothelial Growth Factor C
XIAP	X-linked Inhibitor of Apoptosis Protein
ZEB	Zinc finger E-Box-Binding Homeobox
